# Supplementary material for: Transient fertilization of a post-Sturtian Snowball ocean margin with dissolved phosphate by clay minerals
Source: Nat Commun. 2023 Dec 18;14:8418. doi: 10.1038/s41467-023-44240-9 (PMC10728154; doi:10.1038/s41467-023-44240-9)
Supplement: Supplementary file 1 — Supplementary Information [file 41467_2023_44240_MOESM1_ESM.pdf]

Supplementary information

Transient fertilization of a post-Sturtian Snowball Ocean margin with dissolved phosphate by clay minerals

Ernest Chi Fru[1*] Jalila Al Bahri[1], Christophe Brosson[1], Abderrazzak El Albani[2], Olabode Bankole[2], Jérémie Aubineau[3], Alexandra Nederbragt[1], Anthony Oldroyd[1], Alasdair Skelton[4], Linda Lowhagen[4], David Webster[4], Wilson Y. Fantong[5], Benjamin JW Mills[6], Lewis J. Alcott[6], Kurt O. Konhauser[7], Timothy W. Lyons[8]

[1]College of Physical and Engineering Sciences, School of Earth and Ocean Sciences, Centre for Geobiology and Geochemistry, Cardiff University, Cardiff CF10 3AT, Wales, UK
[2]Université de Poitiers UMR 7285-CNRS, Institut de Chimie des Milieux et Matériaux de Poitiers - 5, rue Albert Turpin (Bât B35) 86073 Poitiers cedex, France
[3]Géosciences Environnement Toulouse, CNRS UMR 5563 (CNRS/UPS/IRD/CNES), Université de Toulouse, Observatoire Midi-Pyrénées, Toulouse, France
[4]Department of Geological Sciences, Stockholm University, 106 91 Stockholm, Sweden
[5]Institute of Geological and Mining Research (IRGM), Box 4110, Yaoundé-Cameroon
[6]School of Earth and Environment, University of Leeds, Leeds LS2 9JT, UK
[7]Department of Earth and Atmospheric Sciences, University of Alberta Edmonton, Alberta, T6G 2E3, Canada
[8]Department of Earth and Planetary Sciences, University of California, Riverside, CA 92521, U.S.A

*Corresponding author: ChiFruE@Cardiff.ac.uk

Sedimentation rates and the iron-based redox proxy

The best estimate is that the BDF succession up to the top of member 3, formed within 250 Kyr following the end of the Sturtian snowball glaciation, according to the following logic:

(1) In the original Snowball Earth hypothesis, cap carbonates were thought to have formed within 10 Kyr, based on instantaneous melting models. However, subsequent magnetic reversals within cap carbonates pointed to longer timeframes, consistent with post-glacial sediment starvation because of seawater level rise[15]. There is a transition from coarse-grained sediments to being very fine grained up the studied sequence, consistent with a rise in seawater level and reduction in detrital loading after the Sturtian glaciation.

(2) Globally, $\delta^{13}C_{carbonate}$ values generally recover from -5‰ towards more positive excursions following the transition from the Sturtian icehouse into the greenhouse interval[5,16]. Similarly, published $\delta^{13}C_{carbonate}$ values for the immediate Bonahaven dolomite formation (BDF) succession upwards to member 3, as are our data, switch towards a more positive $\delta^{13}C_{carbonate}$ values immediately above the PATF.

(3) The abrupt shift of $\delta^{13}C_{carbonate}$ to up to +10‰ in member 4 in the BDF is far more extreme than any reported worldwide, leading some workers to equate this to the "Keele Peak" in Canada. However, the occurrence of member 4 halfway up the Sturtian post-Snowball interval towards the Marinoan Snowball Earth poses a problem, particularly because member 4 is estimated to be older than the Keele Peak. Although no explanation for the anomalous positive $\delta^{13}C_{carbonates}$ value was given, Fairchild interpreted member 4 in a PhD thesis as a supratidal dolomitic deposit. Therefore, the top of member 3 in the BDF lies within the bounds of the timeframe when the negative $\delta^{13}C_{carbonates}$ values were yet to consistently rise towards the strong positive values recorded in member 4.

(4) In the absence of radiometric dates for the BDF, we use unique cyclostratigraphic constraints in the widely accepted classic Sturtian Snowball Earth succession in Svalbard[4], to estimate sedimentation rates for the BDF. Here, the $\delta^{13}C_{carbonate}$ values > -5‰ are consistently recorded up to ~10 m in the immediate post-glacial section which terminates in a shale facies succession. Within this succession, 0.5 m thick precessional cycles estimated to represent 18 Kyr in duration are linked to 6-8 Myr interglacials[17]. To a first approximation, the 10 m thick succession above which $\delta^{13}C_{carbonate}$ switches to ≥ -5‰ values, formed over an estimated 200-250 Kyr interval, is related to the time of deposition of the 300 m thick BDF unit up to member 3, based on stratigraphical correlation and similar and consistent ≤-5‰ $\delta^{13}C_{carbonate}$ values. This suggests an estimated 30 times faster sedimentation rate for the BDF compared to the Svalbard succession, accounted for by syndepositional faulting and basin subsidence associated with the Dalradian Supergroup.

This has implications for the accuracy of the iron-based redox proxy because sedimentation rates of up to 0.0077 m yr^{-1} are reported to falsify its sensitivity and accuracy[18-20]. The above calculations suggest a maximum sedimentation rate of 0.0015 m yr^{-1} for the BDF up to member 3, which is 4× slower than the rate expected to strongly interfere with the accuracy of the iron-based redox proxy[18-20].

Moreover, evidence suggests that depositional rates during the formation of the BDF sequence, switched to a slower pace because of global seawater transgression slowing down sediment supply to the basin. Hence if anything, the iron-based proxy is expected to become more accurate in their prediction of bottom seawater oxygenation at this time, as false oxygenation trends can be produced during rapid sediment accumulation[18-20]. The signal remains consistently anoxic during the deposition of the 1.1 km thick PATF succession, whose sedimentation rates were likely much faster than for the BDF. Moreover, throughout the sequence, the redox signal appears unaffected by carbonate concentration; staying consistently anoxic or oxic, depending on the interval, regardless of whether carbonate concentrations varied from near 0.0 wt.% to 50 wt.%.

Supplementary Figures

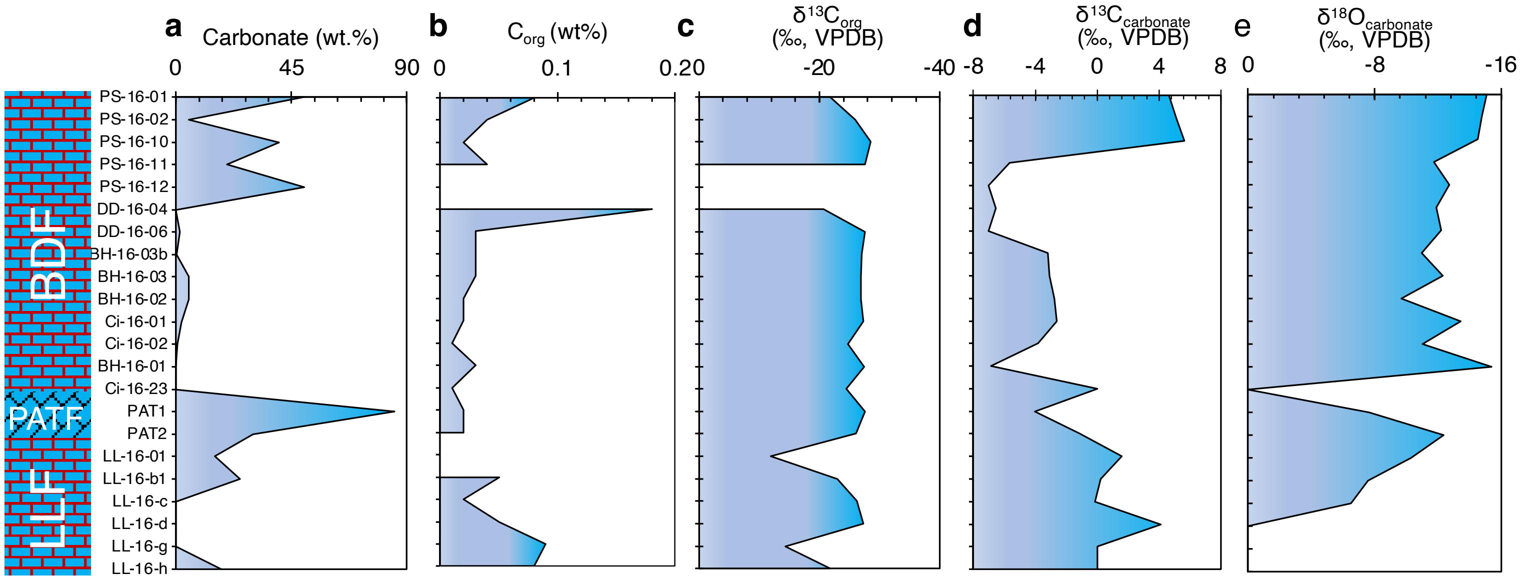

Fig. S1 | Carbon systematics for representative samples plotted along sequence stratigraphy. **(a)** Carbonate concentration. **(b)** C_{org} concentration. **(c)** Table carbon isotope ratios in organic carbon. **(d)** Carbon isotope ratios in carbonates. (e) Oxygen isotope ratios in carbonates. BDF, Bonahaven Dolomite Formation (BDF). PATF, Port Askaig Tillite Formation. LLF, Lossit Limestone Formation. VPDB, Vienna Pee Dee Belemnite. PAT1 and PAT2 are samples collected at Port Askaig.

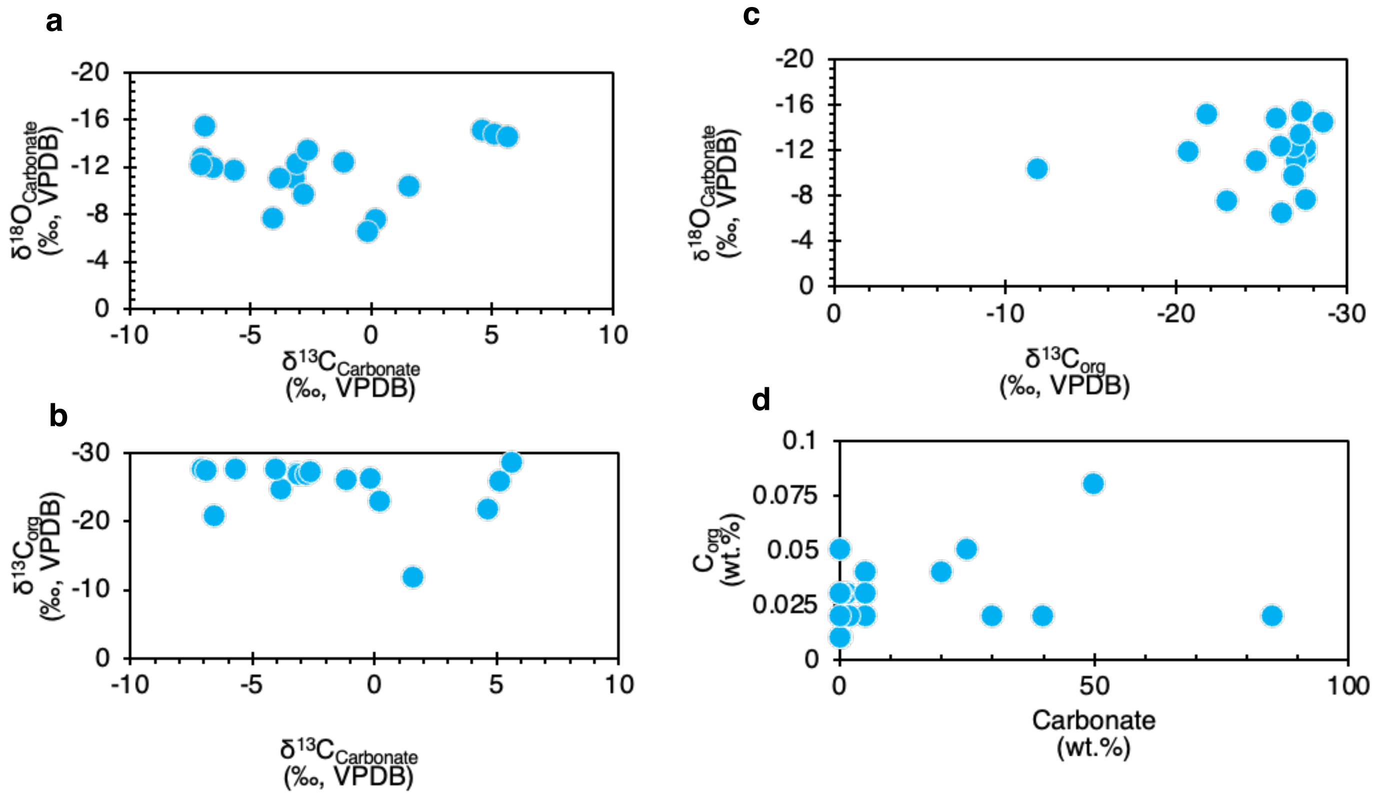

Fig. S2 | (a) $\delta^{18}O_{carbonates}$ versus $\delta^{13}C_{carbonate}$ cross plot. $\delta^{18}O_{carbonates}$ values are spread between -15.09‰ up to -6.48‰, averaging -11.66±2.26‰. Average $\delta^{13}C_{carbonates}$ values of -2.04±4.1‰ range from as low as -7.02‰ and up to +5.64‰. **(b)** $\delta^{13}C_{org}$ versus $\delta^{18}O_{carbonates}$ cross plots. $\delta^{13}C_{org}$ values are typically photosynthetic, ranging from -11.87‰ to -28.57‰, with an average of -24.51±4.36‰. **(c)** $\delta^{18}O_{carbonate}$ versus $\delta^{13}C_{org}$ cross plot. **(d)** C_{org} versus carbonate content cross plot. Carbonate concentrations in the sample suite range from 0.01 wt.% and up to 85 wt.%, averaging 17.6±23.45 wt.% across sequence stratigraphy. C_{org} values average 0.044±0.039 wt.% and range from 0.01 to 0.18 wt.%. The data show that the highest carbonate concentrations are recorded in dolomite-rich samples further up from the interval immediately following the deglaciation in the Bonahaven Dolomite Formation and in the underlying pre-glacial Lossit Limestone Formation. The lowest carbonate concentrations are encountered in the immediate post-glacial deposits. Organic carbon concentrations are generally low across sequence stratigraphy. They range from 0.02-0.08 wt%, averaging 0.038 wt%. No correlation was found between the different variables, as common for Proterozoic C_{org} and carbonate concentrations. The absence of correlation between $\delta^{13}C_{carbonates}$ and $\delta^{18}O_{carbonates}$ is interpreted to suggest limited diagenetic alteration of the primary $\delta^{13}C_{carbonates}$ signal[1,2]. Broadly, the data are consistent with primary C signatures that have been correlated world-wide to the Sturtian Snowball deposits[3-7].

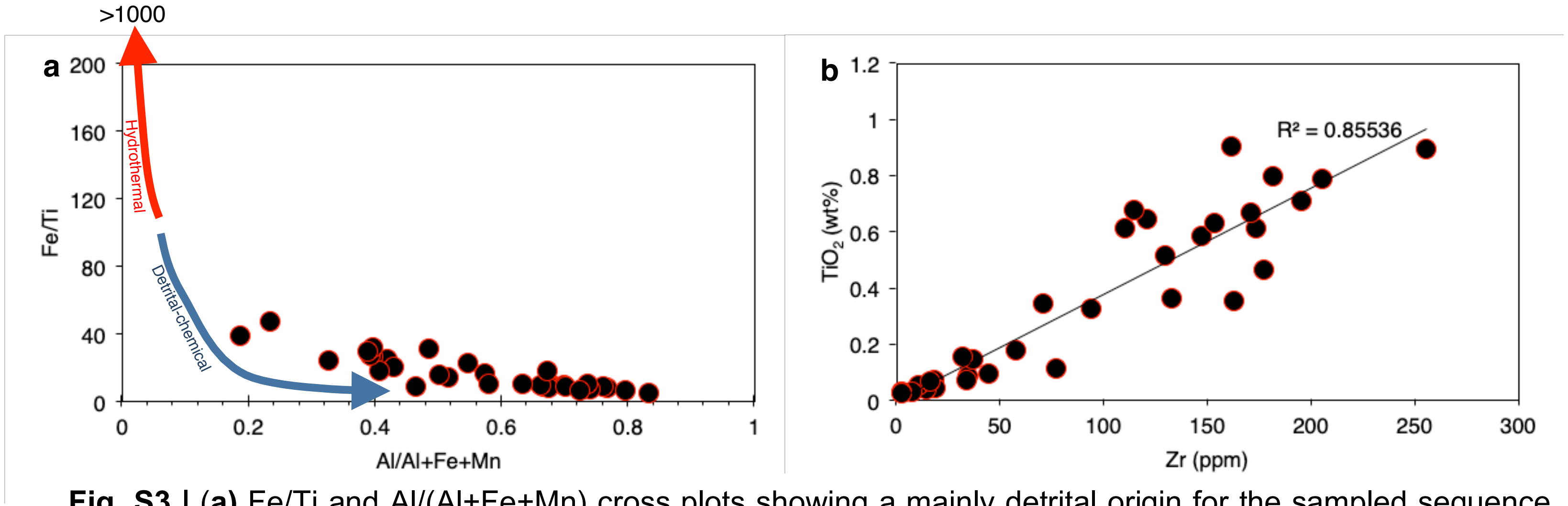

Fig. S3 | (a) Fe/Ti and Al/(Al+Fe+Mn) cross plots showing a mainly detrital origin for the sampled sequence. Red arrow=hydrothermal origin (cut off minimum threshold at 100≥Fe/Ti>1000). Blue arrow shows continuous gradation towards mixed chemical+detrital to pure chemical sediments[9]. **(b)** Relationship between TiO_2 and Zr, showing that the analyzed rocks have a strong detrital origin.

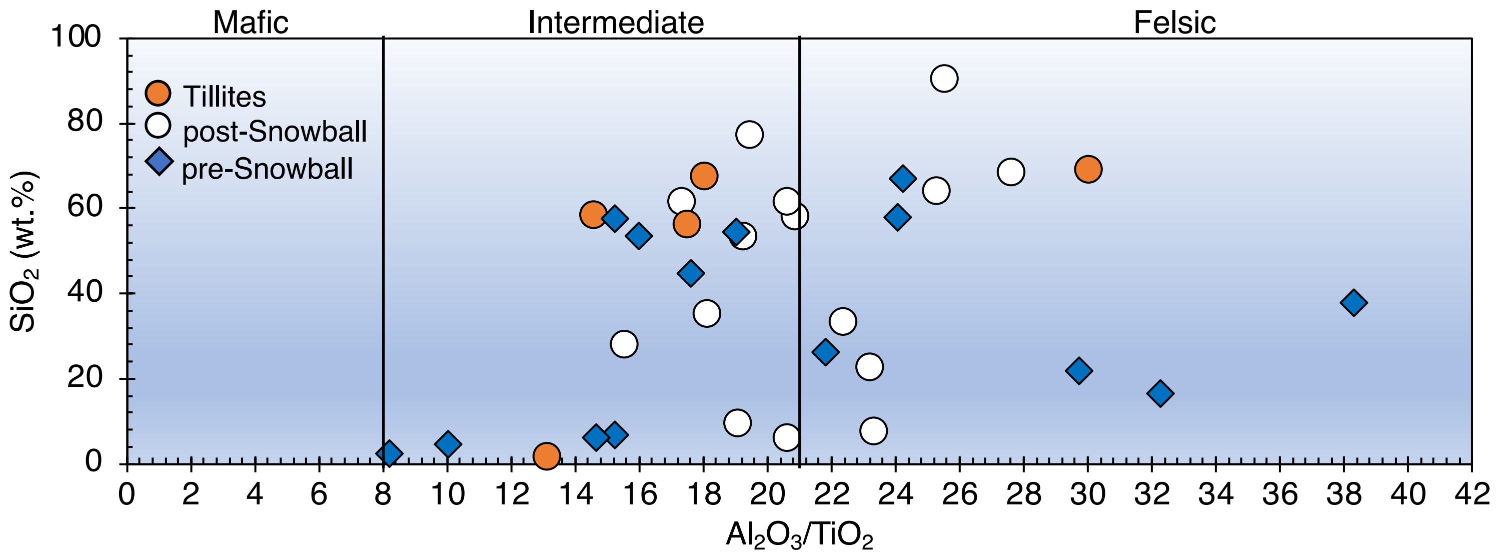

Fig. S4 | SiO_2 versus Al_2O_3/TiO_2 ratios across lithostratigraphy. The distribution suggests the source provenance for the studied lithologies were mainly from intermediate-to-felsic rocks. In weathered igneous rocks, Al is hosted largely by mica and clays and residual feldspars, whereas Ti is hosted primarily by mafic minerals, including pyroxene, biotite, ilmenite. Therefore, the Al_2O_3/TiO_2 ratio was used to broadly estimate the source rock composition, where Al_2O_3/TiO_2 ratios < 8, 8-21 and >21, approximate a mafic, intermediate igneous and felsic igneous source rock composition, respectively[8].

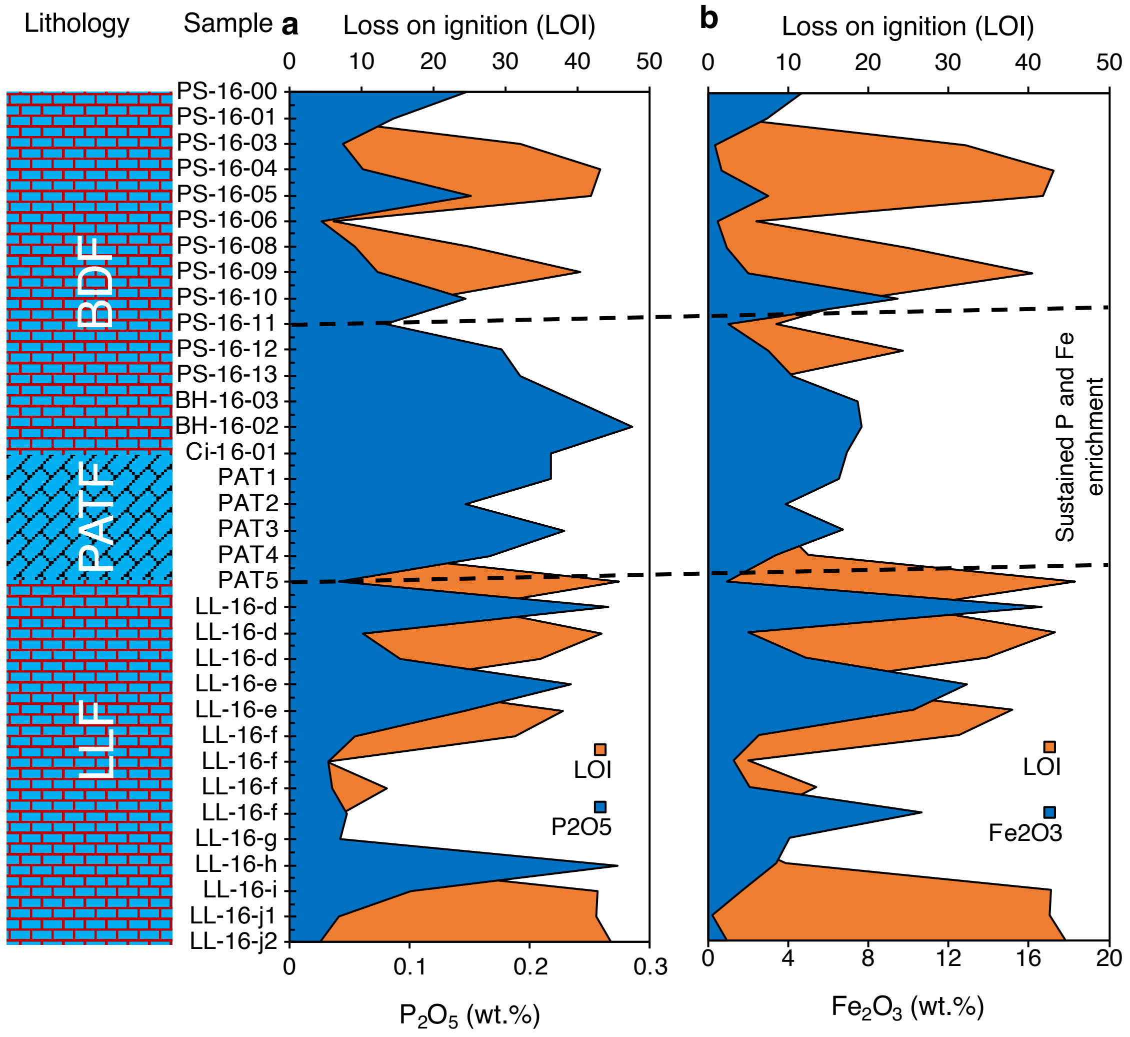

Fig. S5 | Graphs showing an inverse oscillatory behaviour between **(a)** LOI versus P_2O_5 and **(b)** LOI versus Fe_2O_3 distribution across the sampled succession. LLF, Lossit Limestone formation. PATF, Port Askaig Tillite Formation. BDF, Bonahaven Dolomite. PAT1 and PAT2 are samples collected at Port Askaig. PAT3-PAT5 represent sample LL-16-a at the top of the LLF that conformably underlines the Port Askaig tillites.

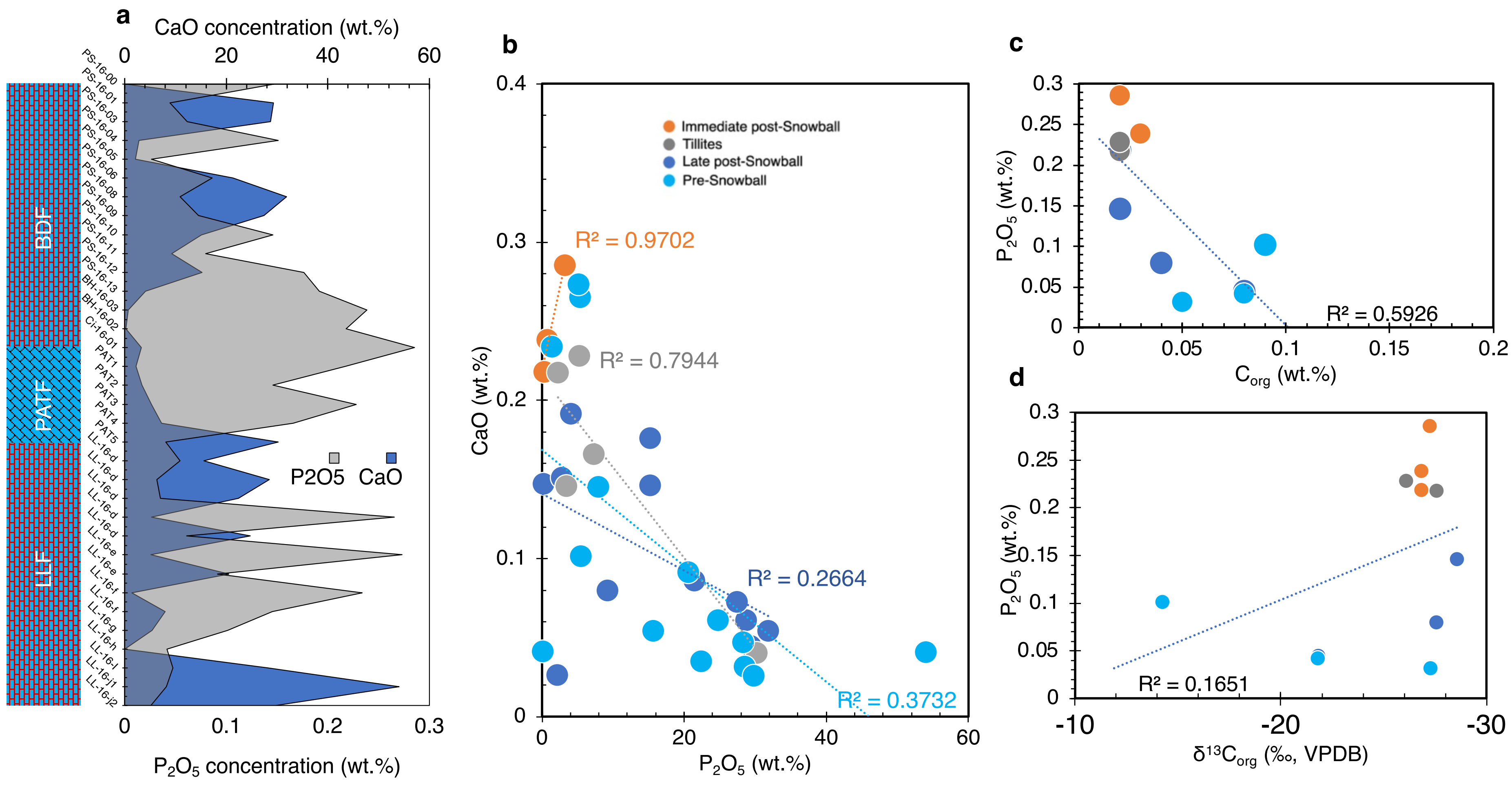

Fig. S6 | Relationship between facies Ca, P, C_{org} and $\delta^{13}C_{org}$ content. (a) CaO versus P_2O_5 distribution along sequence stratigraphy. **(b)** CaO versus P_2O_5 scatter plot. **(c)** Scatter plot for representative data available for P_2O_5 and C_{org} **(d)** Scatter plot for representative data available for P_2O_5 and $\delta^{13}C_{org}$. PAT1 and PAT2 are samples collected at Port Askaig. PAT3 and PAT5 represent sample LL-16-a at the top of the LLF that conformably underlines the Port Askaig tillites.

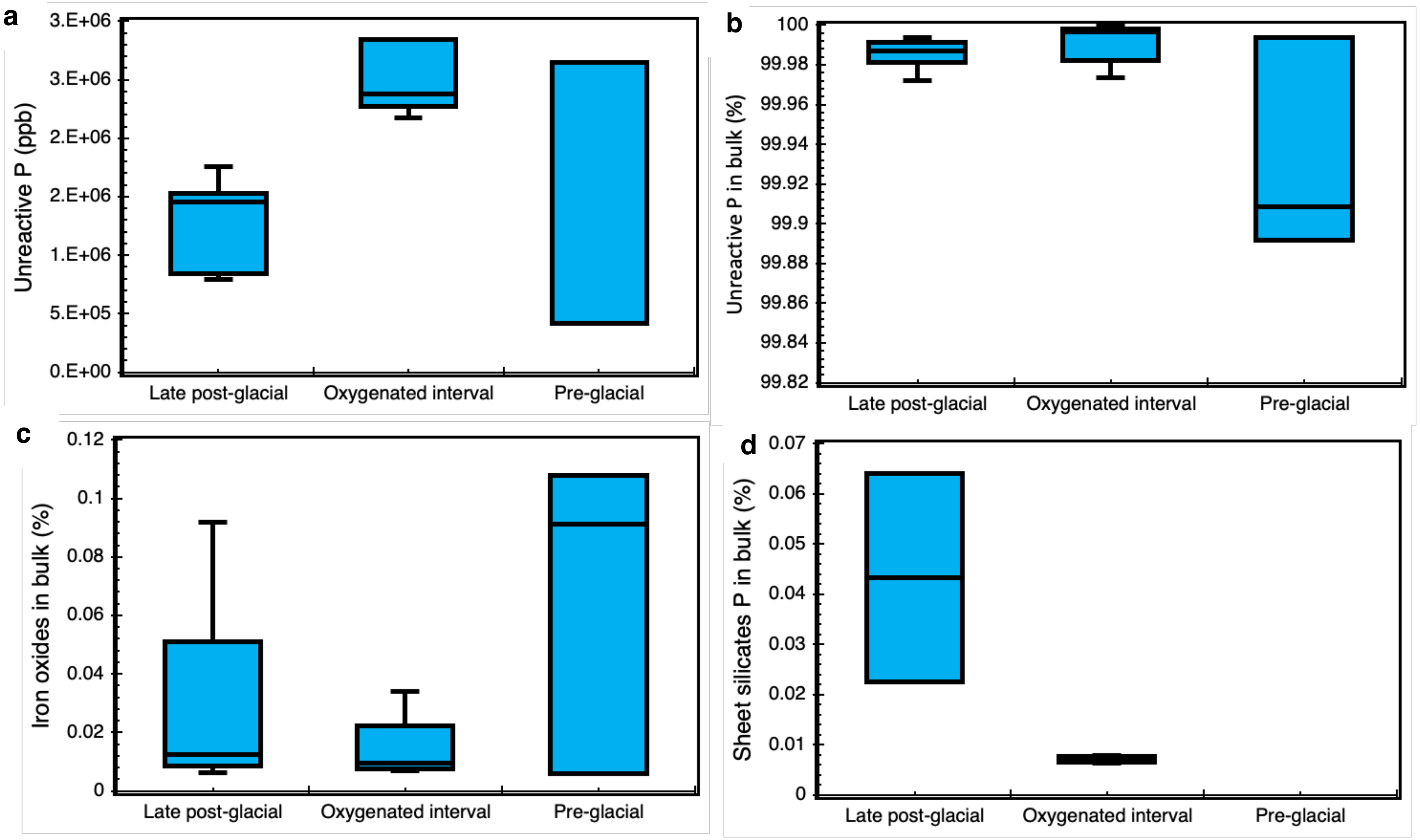

Fig. S7 | Box and whisker plots showing P distribution in various mineral phases relative to bulk P concentrations (P_2O_5) in low P_2O_5 pre-Snowball Lossit Limestone Formation and immediate post-Snowball high P_2O_5 and late post-snowball low P_2O_5 intervals. Bulk unreactive P was calculated by deducting the concentrations of highly reactive iron oxide-bound P and sheet silicate (phyllosilicate) associated P from total bulk measurements for corresponding samples**. (a)** Total unreactive P in ppb. **(b)** % unreactive P in bulk**. (c)** % Fe oxide-bound P in bulk. **(d)** % sheet silicate P in bulk. Centre line = median value; whiskers = minimum and maximum values; box limits = lower and upper quartiles.

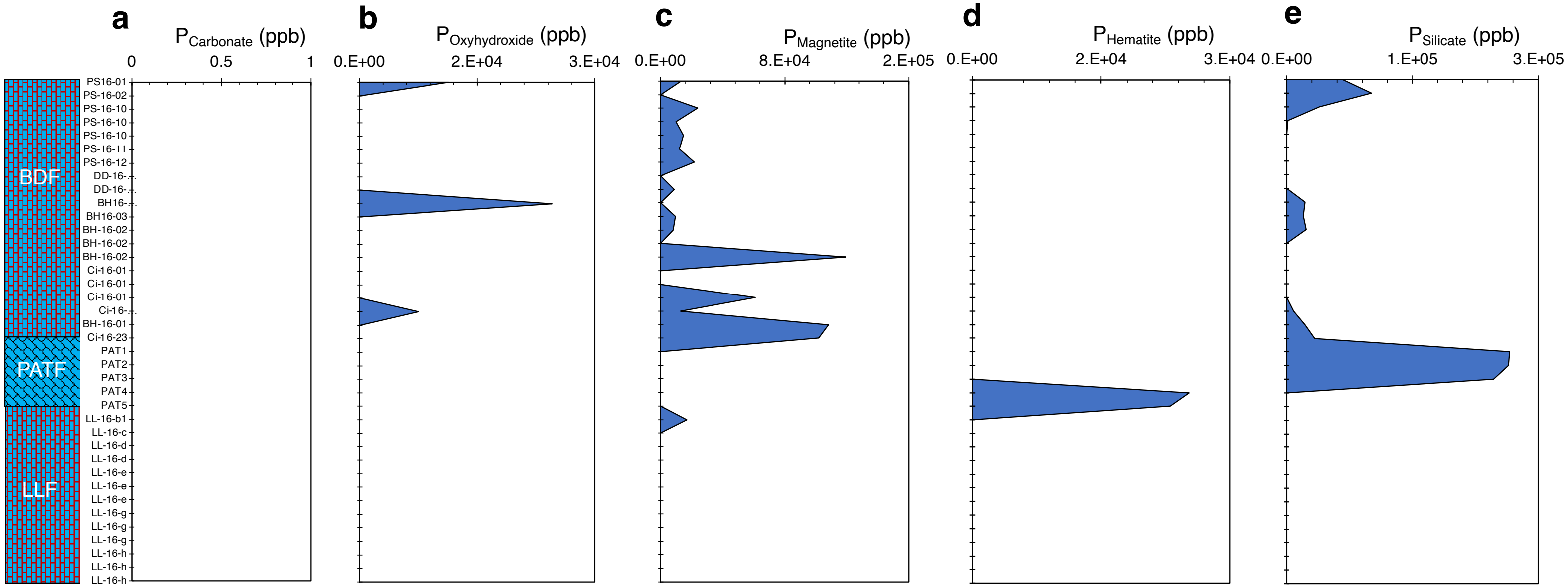

Fig. S8 | Chemically extractable P associated with iron oxides and phyllosilicates (sheet silicates) phases. **(a)** Carbonate associated P. **(b)** Fe oxyhdr(oxide) associated P. **(c)** Magnetite associated P. **(d)** Hematite associated P. **(e)** Sheet silicate associated P. Points were there are no data imply values were below the analytical detection limit of ~5 ppb and must therefore not be interpreted to imply absence. The trends are considered representative of true P distribution, considering that all samples were normalized to the same procedural treatment and analysis. P-oxyhydr(oxides) represent extractions that include goethite, akageneite and some hematite. LLF, Lossit Limestone formation. PATF, Port Askaig Tillite Formation. PAT, Port Askaig Tillites. BDF, Bonahaven Dolomite.

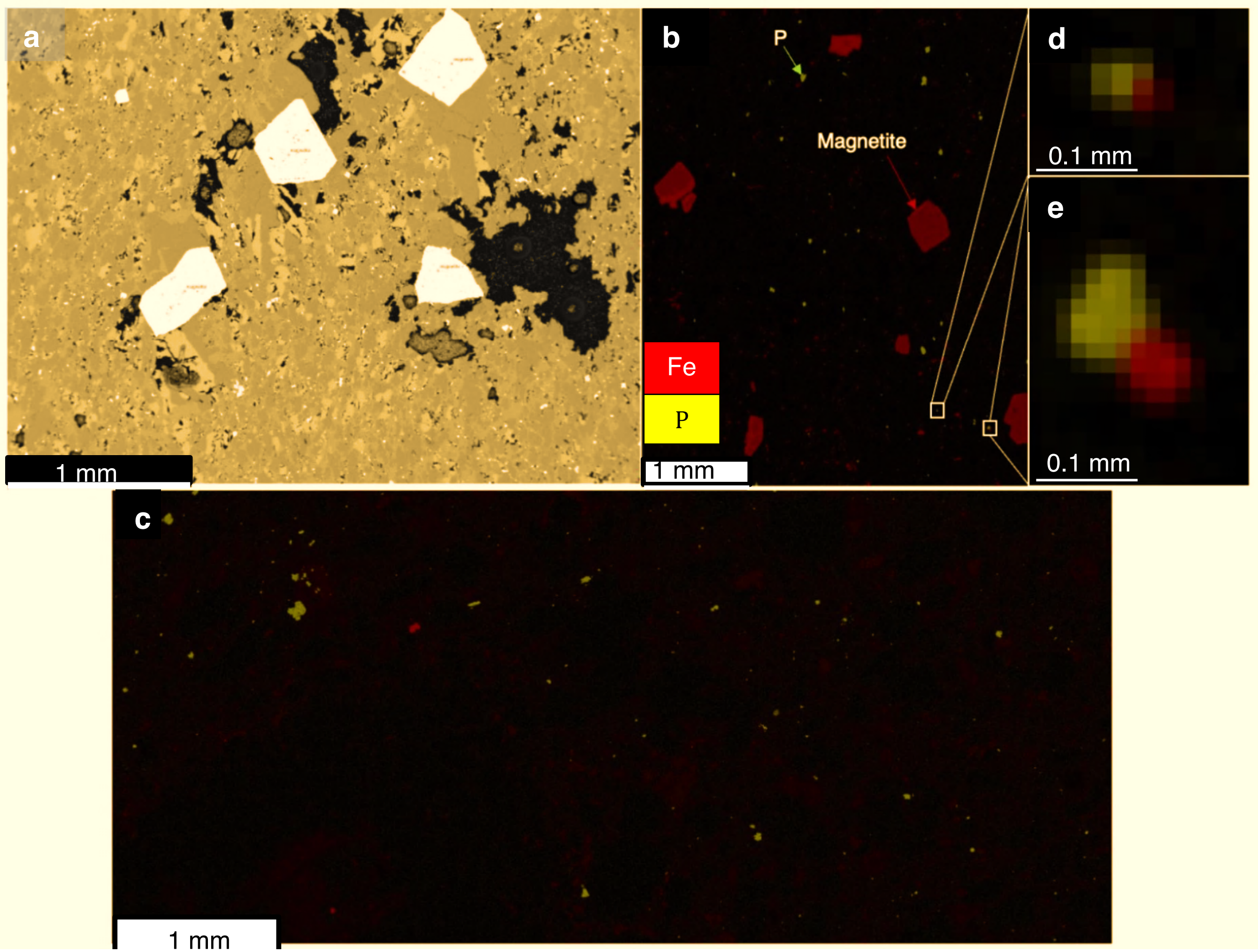

Fig. S9 | Examples of back scatter electron (BSE) SEM and SEM-EDS mineral maps showing the distribution of Fe and P in a representative tillite and immediate post-Snowball sample. **(a)** BSE photomicrograph showing angular and subangular white iron oxide crystals identified as putative magnetite phases in the BH-16-02 immediate post-Snowball sample. **(b)** SEM photomicrograph showing association between magnetite particles and P. **(c-d)** Magnified particles from panel b showing co-occurrence of Fe and P in the immediate post-Snowball samples. **(e)** SEM photomicrograph showing the distribution of Fe and P in a tillite sample. Note the absence of the large blocky magnetite grains in the immediate post-Snowball sample in the tillites sample.

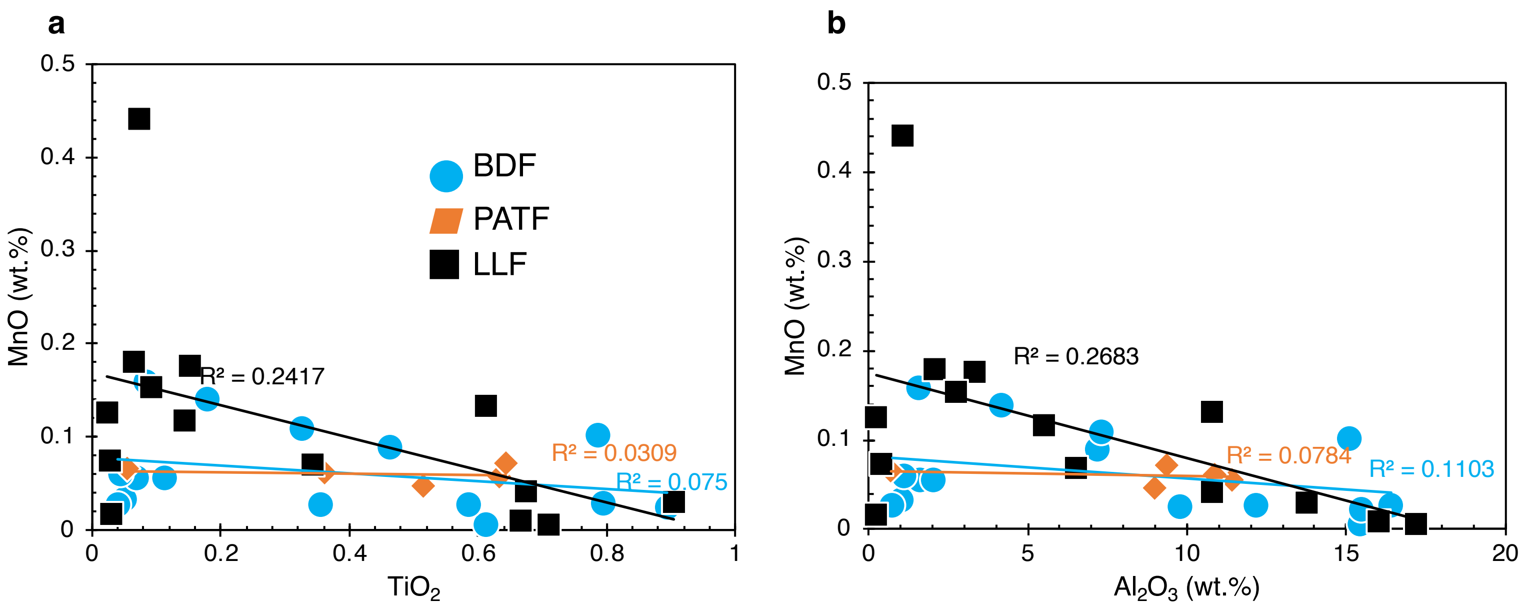

Fig. S10 | MnO versus TiO_2 **(a)** and Al_2O_3 **(b)** cross plots. LLF, Lossit Limestone formation. PATF, Port Askaig Tillite Formation. BDF, Bonahaven Dolomite Formation.

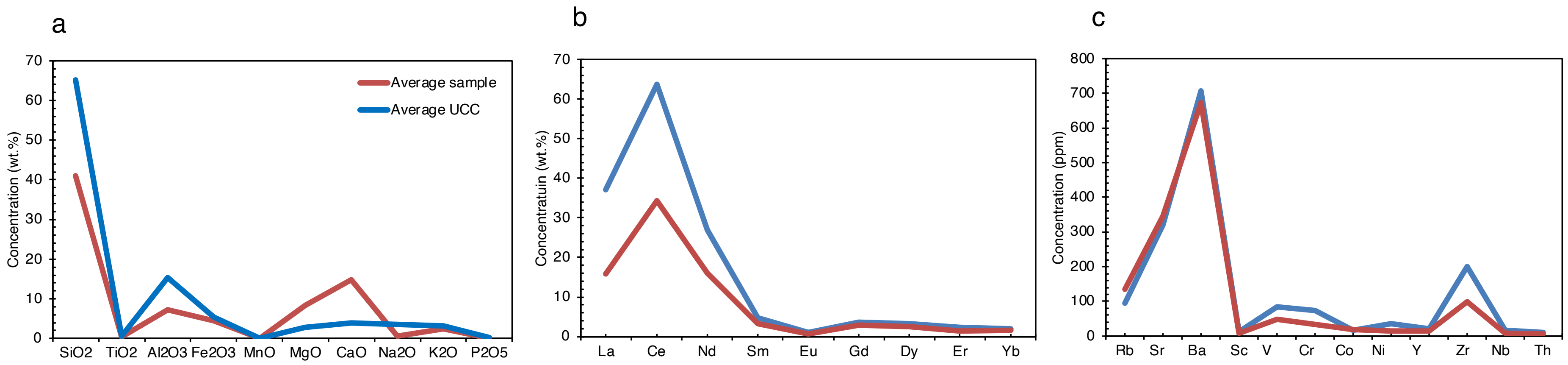

Fig. S11 | Average Upper Continental Crust (UCC) concentrations[ref. 10] for **(a)** major oxides (MO), **(b)** rare earth elements (REE) and **(c)** trace elements (TE) plotted against average sample values. The samples are relatively depleted in MO, REE and TEs relative to UCC, but show similar trends to UCC, suggesting a strong UCC provenance for the sediments. Calcium isotopes have suggested a large flux of contiental materials to the sea at the end of Snowball glaciations[11], supported by Li isotopes indicative of CO_2-induced silicate weathering[12-14].

Supplementary Tables

Table S1 | Sample description.

Locality	Coordinates	Formation and Stratigraphy
**Port an t-Struthain**		
PS-16-01	N55°55.499' W006°11.332'	Bonahaven Dolomite, Member 4 metapelite
PS-16-02	N55°55.500' W006°11.331'	Bonahaven Dolomite, Member 4 Semi metapelite
PS-16-03	N55°55.460' W006°11.331'	Bonahaven Dolomite, Member 4, Middle of the sandwich cream dolostone
PS-16-04	N55°55.528' W006°11.216'	Bonahaven Dolomite, Member 4, cream dolostone
PS-16-05	N55°55.434' W006°11.230'	Bonahaven Dolomite, Member 3, iron rich dolostone
PS-16-06	N55°55.432' W006°11.199'	Bonahaven Dolomite, Member 2, quartzite
PS-16-10	N55°55.489' W006°11.184'	Bonahaven Dolomite, Low member 4 or high member 3
PS-16-11	N55°55.542' W006°10.769'	Bonahaven Dolomite, Member 3 Muddy dolostone
PS-16-12	N55°55.539' W006°10.644'	Bonahaven Dolomite, Member 3 Dolomitic mudstone
**Port a'Chotain**		
PC-16-01a	N55°55.726' W006°10.069'	Bonahaven Dolomite, Member 4, Foliated
PC-16-01b	N55°55.726' W006°10.069'	Bonahaven Dolomite, Member 4, Massive
PC-16-02	N55°55.672' W006°09.806'	Bonahaven Dolomite, Member 4, Dolostone
PC-16-03	N55°55.760' W006'09.918'	Bonahaven Dolomite, Member 3 (just under member 4), Dolostone
**Bagh an da Dhoruis**		
DD-16-04	N55°56.005' W006°09.425'	Bonahaven Dolomite, Member 3 Muddy dolostone under stromatolite
DD-16-06	N55°56.002' W006°09.072'	Bonahaven Dolomite, Member 3 Muddy dolostone
DD-16-07	N55°55.973' W006°08.664'	Bonahaven Dolomite, Member 4
**Caol Isla**		

Ci-16-01	N55°51.375' W006°06.570'	Bonahaven Dolomite, Member 1 unit 1 Metapelite mudstone biotite
Ci-16-02	N55°51.431' W006°06.555'	Bonahaven Dolomite, Member 1 unit 4 (very base)
Ci-16-23	N55°51.460' W006°06.531'	Bonahaven Dolomite, Top member 4 mudstone
Ci-16-03b	N55°51.460' W006°06.531'	Bonahaven Dolomite, Base member 5 dolostone
**Bonahaven**		
BH-16-01	N55°52.704' W006°07.118'	Bonahaven Dolomite, Member 1 unit 1 Metapelite with carbonate pebbles
BH-16-02	N55°52.750' W006°07.156'	Bonahaven Dolomite, Member 1 unit 4 Mud and siltstone. Unmetamorphosed
BH-16-03	N55°52.763' W006°07.168'	Bonahaven Dolomite, Member 1 unit 5 Mud layer growing over mica cement. Unmetamorphosed
BH-16-03b	N55°52.762' W006°07.177'	Bonahaven Dolomite, Member 1 unit 5 Mud layer
BH-16-04	N55°52.987' W006°07.309'	Bonahaven Dolomite, Member 3 Stromatolitic dolostone
**Lossit**		
LL-16-k	N55°48.293' W006°07.379'	Lossit Limestone
LL-16-01	N55°48.194' W006°07.781'	Lossit Limestone
LL-16-j	N55°48.317' W006°07.464'	Lossit Limestone, Grey limestone
LL-16-j(b)	N55°48.323' W006°07.505'	Lossit Limestone, Oolitic dolostone
LL-16-i	N55°48.374' W006°07.525	Lossit Limestone, Stromatolitic
LL-16-h	N55°48.383' W006°07.655'	Lossit Limestone, Slate
LL-16-g	N55°48.412' W006°07.732'	Lossit Limestone, Muddy limestone
LL-16-c	N55°48.451' W006°07.670'	Lossit Limestone, Limestone
LL-16-d	N55°48.467' W006°07.698'	Lossit Limestone, Muddy layer in metacarbonate (mudstone)
LL-16-e	N55°48.435' W006°07.823'	Port Askaig tillite, Muddy layer in diamictite
LL-16-f	N55°48.401' W006°07.863'	Port Askaig tillite, Magnetic sandstone interlayered by dolostone diamictite
LL-16-b2	N55°48.445' W006°07.609'	Port Askaig tillite, Dolostone diamictite
LL-16-b1	N55°48.442' W006°07.605'	Port Askaig tillite, Mudstone diamictite. Contain a carbonate clast
LL-16-a	N55°48.520' W006°07.553'	Port Askaig tillite, Muddy tillite
LL-16-a2	N55°48.519' W006°07.535'	Port Askaig tillite, Clast of dolostone in tillite

**Creagan Loisgte**		
CL-16-01	N55°49.262' W006°06.787'	Port Askaig tillite, Member 3, Dolostone
CL-16-02	N55°49.353' W006°06.695'	Port Askaig tillite, Member 3, dolostone, the second diamictite
CL-16-03	N55°49.370' W006°06.747'	Port Askig tillite, Top member 3, Metapelit

Table S2 | Semi-quantitative bulk XRD mineralogical composition for representative samples measured in weight percent (wt.%). PAT1 and PAT2 are samples collected at Port Askaig. PAT3 represents sample LL-16-a at the top of the LLF that conformably underline the Port Askaig tillites. nd=not detected.

Samples	Quartz	Illite	Calcite	Albite	Kaolinite	Dolomite	Microcline	Pyrite	Hematite
**PS-16-11**	33	9	9	7	nd	40	nd	2	nd
**PS-16-12**	36	8	nd	nd	2	50	nd	nd	nd
**PS-16-13**	50	11	nd	11	nd	18	7	3	nd
**BH-16-03**	53	23	nd	8	nd	13	nd	nd	3
**BH-16-02**	68	17	nd	11	2	nd	nd	nd	2
**Ci-16-01**	53	35	nd	nd	nd	nd	nd	nd	nd
**PAT1**	41	5	5	46	2	1	nd	nd	nd
**PAT2**	48	14	7	25	4	2	nd	nd	nd
**PAT3**	54	16	nd	7	2	21	nd	nd	nd
**LL-16-g**	55	18	nd	12	1	nd	nd	nd	1
**LL-16-i**	5	1	nd	3	nd	91	nd	nd	nd
**LL-16-j**	1	nd	1	nd	nd	98	nd	nd	nd

Table S3 | Trace and rare earth elements (ppm) and major elements (wt.%) data for representative samples across the sampled section. LD, lower than detection limit. PATF, Port Askaig Tillite Formation.

	Bonahaven Dolomite Formation (BDF)														
	PS-16-00	PS-16-03	PS-16-04	PS-16-05	PS-16-06	PS-16-01	PS-16-08	PS-16-09	PS-16-10	PS-16-11	PS-16-12	PS-16-13	Ci-16-01	BH-16-02	BH-16-03
SiO_2	64.3	6.2	7.7	68.6	90.6	22.9	35.5	9.7	28.3	77.4	33.6	58.2	61.6	61.7	53.5
TiO_2	0.61	0.05	0.07	0.35	0.04	0.18	0.11	0.08	0.46	0.04	0.33	0.58	0.79	0.89	0.79
Al_2O_3	15.4	1.0	1.6	9.8	1.1	4.2	2.0	1.6	7.2	0.8	7.3	12.2	16.4	15.5	15.1
Fe_2O_3	4.7	0.4	0.7	3.0	0.5	3.0	1.0	2.0	9.5	1.0	3.0	4.2	6.9	7.5	7.6
MnO	0.006	0.033	0.056	0.027	0.060	0.140	0.056	0.160	0.090	0.027	0.109	0.027	0.028	0.024	0.102
MgO	3.00	20.07	18.70	3.37	1.24	13.20	1.67	17.16	8.90	1.58	10.98	3.80	2.20	2.14	3.80
CaO	0.14	29.33	28.73	2.78	2.06	21.37	31.83	27.43	15.15	9.19	15.22	4.03	0.25	0.66	3.21
Na_2O	0.09	0.43	0.39	0.08	0.17	0.75	0.19	0.54	0.70	0.01	0.04	1.36	1.90	0.87	0.70
K_2O	7.34	0.23	0.47	4.58	0.46	1.56	0.83	0.46	2.95	0.30	3.50	4.81	5.81	6.18	6.42
P_2O_5	0.147	0.045	0.061	0.151	0.026	0.086	0.054	0.073	0.146	0.080	0.176	0.191	0.218	0.238	0.285
LOI	3.12	43.10	41.75	6.04	3.12	32.07	25.03	40.39	19.38	8.52	24.32	9.38	2.84	3.60	7.37
Rb	287	<LD	<LD	131	<LD	26	44	<LD	85	<LD	85	139	238	259	283
Sr	6.2	255	414	31.3	27.4	301	1172	315	177	48.1	116	60.2	39	72	80
Ba	424	54.3	1492	505	539	185	10120	220	304	49.4	249	644	670	449	383
Sc	12.5	0.8	1.2	5.6	0.7	3.2	1.6	1.2	7.1	0.6	5.8	10.4	14.7	14.4	16.3
V	84.3	6.6	9.3	38.4	7.1	22.7	15.0	15.5	61.4	6.4	54.7	70.7	80.8	103	91.2
Cr	52.6	5.2	6.4	33.4	33.2	19.3	12.1	8.7	39.0	7.2	33.0	60.3	61.1	61.5	48.1
Co	9.1	4.5	5.6	26.8	85.4	5.2	19.3	3.8	25.7	54.1	22.0	30.9	27.0	14.0	15.8
Ni	9.2	2.6	3.3	10.9	2.5	7.8	3.0	3.6	54.5	7.1	16.1	29.3	30.3	25.5	31.5
Y	21.4	4.9	9.1	15.5	3.6	18.2	23.3	10.2	27.9	6.3	21.3	19.7	19.0	36.9	34.4
Zr	173	12.0	18.5	163	18.8	57.7	76.9	34.7	178	14.7	94.1	147	181	256	205
Nb	21.9	0.3	1.5	7.9	1.0	3.2	2.8	2.4	13.4	1.2	7.3	13.2	22.6	28.8	25.7
La	39.2	3.6	4.4	17.7	5.2	6.7	7.6	5.3	18.0	4.9	12.2	25.6	18.5	54.5	27.1
Ce	79.7	7.4	9.1	38.1	10.5	14.8	16.4	12.6	34.8	8.6	25.1	53.7	76.8	115	57.3
Nd	36.6	3.7	5.6	17.7	4.8	8.6	8.6	5.1	17.5	5.1	13.2	24.0	19.4	54.8	26.7
Sm	6.4	1.0	1.1	3.4	0.8	2.2	2.7	1.3	3.7	1.0	3.1	4.6	4.3	10.7	5.3
Eu	1.19	0.20	0.32	0.62	0.28	0.74	1.60	0.37	0.70	0.25	0.63	0.75	0.70	1.66	1.01
Gd	5.1	0.5	1.2	3.0	0.7	2.9	3.2	1.5	4.5	1.2	3.3	4.0	4.2	7.7	5.7
Dy	3.8	0.8	1.3	2.4	0.6	3.2	3.6	1.7	4.8	1.0	3.4	3.1	3.8	6.4	5.6
Er	1.9	0.7	0.5	1.6	0.1	1.8	2.0	1.3	2.8	0.4	2.1	2.1	1.6	3.5	3.1
Yb	2.46	0.39	0.76	1.56	0.27	1.54	2.27	0.77	2.97	0.59	1.85	2.17	2.23	3.90	3.54
Th	10.0	1.1	1.7	5.7	0.8	2.5	3.0	1.6	8.8	1.4	5.1	9.1	21.9	17.8	14.0

	Port Askaig Tillites																		
	PATF		Lossit Limestone Formation																
	Port Askaig tillites	Port Askaig tillites	LL-16-a1	LL-16-a2	LL-16-a3	LL-16-d	LL-16-d	LL-16-d	LL-16-e	LL-16-e	LL-16-f	LL-16-f	LL-16-f	LL-16-g	LL-16-h	LL-16-f	LL-16-i	LL-16-j1	LL-16-j2
SiO_2	67.7	69.3	58.7	56.3	1.7	37.8	6.8	21.8	44.8	16.6	26.2	57.7	54.4	58.0	67.2	53.7	6.1	2.3	4.6
TiO_2	0.63	0.36	0.64	0.51	0.05	0.14	0.03	0.09	0.61	0.07	0.15	0.90	0.34	0.67	0.71	0.67	0.07	0.03	0.02
Al_2O_3	11.4	10.9	9.4	9.0	0.7	5.5	0.4	2.7	10.8	2.1	3.3	13.8	6.5	16.0	17.2	10.8	1.1	0.2	0.2
Fe_2O_3	6.6	3.9	6.7	3.4	1.0	2.6	1.3	2.1	16.7	2.0	4.9	12.9	10.2	4.1	3.4	10.7	1.8	0.3	0.9
MnO	0.057	0.062	0.072	0.047	0.066	0.117	0.074	0.154	0.133	0.181	0.177	0.030	0.070	0.010	0.006	0.042	0.441	0.018	0.126
MgO	2.34	1.23	4.23	5.80	20.76	10.49	19.69	15.10	5.68	16.62	12.19	2.45	5.03	1.34	1.48	4.83	19.35	0.80	20.14
CaO	2.23	3.35	5.20	7.20	30.19	15.59	28.43	22.33	5.25	24.66	20.54	1.37	7.92	5.36	<LD	5.14	28.29	54.01	29.79
Na_2O	2.19	4.03	0.82	1.52	0.01	1.12	0.01	0.02	0.05	0.08	1.02	0.09	0.45	1.25	1.14	0.08	0.36	0.00	0.00
K_2O	3.20	1.70	3.45	2.76	0.13	1.25	0.14	0.70	3.63	0.03	0.17	5.01	1.78	5.00	5.83	4.36	0.20	0.06	0.02
P_2O_5	0.217	0.146	0.228	0.166	0.041	0.054	0.032	0.035	0.265	0.061	0.092	0.234	0.145	0.101	0.042	0.273	0.047	0.041	0.026
LOI	3.53	3.87	9.35	12.50	45.71	24.76	43.33	34.71	10.85	37.91	31.29	5.12	13.47	7.23	2.87	9.74	42.68	42.53	44.59
Rb	93	43	101	81	<LD	27	<LD	<LD	<LD	<LD	<LD	177	44	178	229	132	<LD	<LD	<LD
Sr	113.8	176	113	157	662	436	308	348	249	696	924	512	587	66.9	37.2	235	277	2387	330
Ba	512	293	511	459	12.3	193	14.7	101	479	6.4	67.4	1559	578	405	768	794	50.2	7.3	0
Sc	11.1	6.7	11.0	9.1	0.6	3.5	0.3	1.7	13.5	1.4	5.2	18.8	8.7	11.4	13.1	12.3	1.0	0.4	0.1
V	82.3	39.6	89.5	80.0	6.0	21.8	6.5	11.5	95.4	8.4	12.8	158	67.0	64.5	64.9	98.4	5.9	2.7	3.0
Cr	57.8	30.0	53.8	46.4	6.2	17.3	3.0	8.0	49.9	13.1	14.8	52.6	28.6	73.5	76.8	50.3	6.4	3.6	3.4
Co	20.9	28.2	26.4	33.5	3.8	7.2	6.4	9.5	11.3	7.1	8.6	8.0	22.6	19.8	13.2	15.2	6.7	2.4	5.8
Ni	27.5	16.9	19.3	27.7	5.0	8.8	3.4	5.2	24.1	4.8	7.0	15.2	9.7	26.7	6.6	17.5	3.0	2.0	2.9
Y	19.4	24.2	14.9	15.5	2.8	9.8	2.5	7.1	17.5	7.8	17.0	15.3	13.1	10.5	14.9	16.6	7.9	2.6	9.8
Zr	154	133	121	130	11.0	37.2	7.8	45	110	16.2	32.4	162	70.6	171	195	115	33.7	2.4	2.4
Nb	15.2	10.6	13.0	9.1	0.9	1.8	0.4	2.0	11.0	1.0	3.2	12.1	2.4	13.4	18.6	17.9	1.1	0.1	0.3
La	21.2	21.4	13.0	17.6	1.9	6.1	2.1	4.2	19.4	3.8	7.5	36.5	14.3	17.3	48.7	20.7	5.9	2.1	3.6
Ce	44.3	45.2	28.3	37.1	5.0	11.5	4.1	10.7	38.9	7.0	19.8	72.7	29.4	33.4	99.0	40.7	13.2	6.3	9.1
Nd	18.8	20.3	14.3	16.5	2.5	7.2	2.7	5.1	20.2	5.1	10.9	32.6	16.5	16.2	49.0	20.1	6.2	1.5	4.7
Sm	3.7	4.4	2.6	3.6	0.3	1.5	1.0	0.8	3.9	0.9	2.2	6.1	3.9	2.8	9.1	3.7	1.4	0.2	0.9
Eu	0.69	0.81	0.70	0.65	0.05	0.33	0.07	0.23	0.83	0.24	0.56	1.21	0.65	0.29	1.65	0.73	0.11	0.02	0.10
Gd	2.8	4.2	2.8	3.1	0.0	1.5	0.4	1.0	3.6	1.1	2.7	4.0	2.5	2.2	6.5	3.0	1.1	0.4	1.3
Dy	2.3	3.9	2.6	2.4	0.5	1.4	0.3	0.9	3.1	1.1	2.7	2.6	2.5	1.9	3.1	2.8	1.2	0.3	1.5
Er	1.1	2.2	1.7	1.7	0.3	1.2	0.4	0.3	2.0	0.5	1.5	1.5	1.4	1.2	1.4	1.6	0.3	0.3	0.6
Yb	1.35	2.80	1.65	1.75	0.23	0.99	0.17	0.63	1.96	0.63	1.52	1.84	1.27	1.54	1.77	1.76	0.64	0.15	0.84
Th	7.0	24.2	3.5	5.4	0.8	1.9	0.2	1.8	5.3	1.4	2.3	7.7	4.2	9.8	12.7	5.5	2.2	0.3	1.4

**Table S4 | ** Bulk iron isotope data for sampled cross section.

Sample	Redox	$\delta^{56/54}Fe$ (‰ IRMM)	2sd	$\delta^{57/54}Fe$ (‰ IRMM)	2sd
PS16-01	Anoxic/ferruginous	0.215405186	0.05467559	0.31888514	0.1368159
PS16-02	Anoxic/ferruginous	-0.18805042	0.05467559	-0.2567931	0.1368159
PS16-10	?/ferruginous	0.119779336	0.05467559	0.2126936	0.1368159
PS16-11	Anoxic/ferruginous	0.140901425	0.05467559	0.19806062	0.1368159
PS16-12	Anoxic/ferruginous	0.515277145	0.05467559	0.75800868	0.1368159
DD16-04	Anoxic/ferruginous	0.275878871	0.05467559	0.41860195	0.1368159
DD16-06	Anoxic/ferruginous	0.075870849	0.05467559	0.1294508	0.1368159
BH16-03b	Oxic/ferruginous	0.394539782	0.05467559	0.51466223	0.1368159
BH16-03	Oxic/ferruginous	0.107515842	0.05467559	0.12859005	0.1368159
BH16-02	Oxic/ferruginous	0.206767384	0.05467559	0.36096346	0.1368159
Ci16-01	Oxic/ferruginous	0.127659478	0.05467559	0.14509852	0.1368159
BH16-01	Oxic/ferruginous	0.18512519	0.05467559	0.29501582	0.1368159
CI16-02	Anoxic/ferruginous	0.02869971	0.05467559	0.00057598	0.1368159
CI16-03b	Anoxic/ferruginous	0.139915033	0.05467559	0.17478635	0.1368159
LL16-a	Anoxic/ferruginous	0.316555954	0.05467559	0.47225133	0.1368159
LL16-b1	Anoxic/ferruginous	-0.36464086	0.05467559	-0.5050093	0.1368159
LL16-01	Anoxic/ferruginous	0.018283823	0.05467559	0.01916605	0.1368159
LL16-c	Anoxic/ferruginous	0.033044669	0.05467559	-0.0394956	0.1368159
LL16-d	Anoxic/ferruginous	0.605696336	0.05467559	0.8714074	0.1368159
LL16-g	Anoxic/ferruginous	0.088431765	0.0873112	0.14120006	0.15361095
LL16-h	Anoxic/ferruginous	0.272097494	0.05467559	0.43754128	0.1368159
LL-16-i	Anoxic/ferruginous	0.209865936	0.05467559	0.33961185	0.1368159

Supplementary References

1. Banner, J.L. & Hanson, G.N. Calculation of simultaneous isotopic and trace element variations during water-rock interaction with applications to carbonate diagenesis. *Geochim. Cosmo. Acta* **54**, 3123–3137 (1990).
2. Derry, L.A. A burial diagenesis origin for the Ediacaran Shuram–Wonoka carbon isotope anomaly. *Earth Plan. Sci. Lett*. **294**, 152–162 (2010).
3. Arnaud, E. & Fairchild, I.J. The Port Askaig Formation, Dalradian Supergroup, Scotland. *Geol. Soc. London Mem.* **36**, 635–642 (2011).
4. Brasier, M.D. & Shields, G. Neoproterozoic chemostratigraphy and correlation of the Port Askaig glaciation, Dalradian Supergroup of Scotland. *J. Geol. Soc. London* **157**, 909–914 (2000).
5. Thomas, C.W., Graham, C.M., Ellam, R.M. & Fallick, A.E. $^{87}Sr/^{86}Sr$ chemostratigraphy of Neoproterozoic Dalradian limestones of Scotland and Ireland: constraints on depositional ages and time scales. *J. Geol. Soc. London* **161**, 229–242 (2004).
6. Fairchild, I.J. et al. Tonian-Cryogenian boundary sections of Argyll, Scotland. *Pre. Res.* **319**, 37–64 (2018).
7. Sawaki Y, et al. $^{87}Sr/^{86}Sr$ chemostratigraphy of Neoproterozoic Dalradian carbonates below the Port Askaig Glaciogenic Formation, Scotland. *Pre. Res.* **179**, 150–164 (2010).
8. Hayashi K-I, Fujisawa H, Holland HD, Ohmoto H. Geochemistry of ~ 1.9 Ga sedimentary rocks from northeastern Labrador, Canada. Geochim. Cosmo. Acta 61, 4115–4137 (1997).
9. Pecoits, E., Gingras, M.K., Barley, M.E., Kappler, A., Posth, N.R. & Konhauser KO. Petrography and geochemistry of the Dales Gorge banded iron formation: Paragenetic sequence, source and implications for palaeo-ocean chemistry. *Pre. Res.* **172**, 163–187 (2009).
10. Rudnick RL, Gao S. Composition of the continental crust. In The Crust, vol. 3 (ed. Rudnick RL). Elsevier, 1-64 ((2003).
11. Kasemann, S.A., Hawkesworth, C.J., Prave, A.R., Fallick, A.E. & Pearson, P.N. Boron and calcium isotope composition in Neoproterozoic carbonate rocks from Namibia: Evidence for extreme environmental change. *Earth and Plan. Sci. Lett*. **231**, 73-86 (2005).
12. Millot, R., Vigier, N. & Gaillardet, J. 2010. Behaviour of lithium and its isotopes during weathering in the Mackenzie Basin, Canada. *Geochim. Cosmochim. Acta* **74**, 3897-3912 (2010).
13. Verney-Carron, A., Vigier, N. & Millot, R. Experimental determination of the role of diffusion on Li isotope fractionation during basaltic glass weathering, *Geochim. Cosmochim. Acta* **75**, 3452-3468 (2011).
14. Vigier, N., Gislason, S.R., Burton, K.W., Millot, R. & Mokadem, F. The relationship between riverine lithium isotope composition and silicate weathering rates in Iceland. *Earth Plan. Sci. Lett*. **28**7, 434-441 (2009).
15. Rooney, A.D., Chew, D.M. & Selby, D.) Re–Os geochronology of the Neoproterozoic–Cambrian Dalradian Supergroup of Scotland and Ireland: Implications for Neoproterozoic stratigraphy, glaciations and Re–Os systematics. *Pre. Res.* **185**, 202–214 (2011).
16. Prave, T., Fallick, A.E., Thomas, C.W. & Graham, C.M. A composite C-isotope profile for the Neoproterozoic Dalradian Supergroup of Scotland and Ireland. *J.*

Geol. Soc. **166**, 845–857 (2009).
17. Fairchild, I.J., et al. The Late Cryogenian Warm Interval, NE Svalbard: Chemostratigraphy and genesis. *Pre. Res.* **281**, 128–154 (2016).
18. Raiswell, R. et al. The iron paleoredox proxies: A guide to the pitfalls, problems and proper practice. *Am. J. Sci*. **318**, 491–526 (2018).
19. Poulton, S.W. & Canfield, D.E. Ferruginous conditions: A dominant feature of the ocean through Earth's history. *Elements* **7**, 107-112 (2012).
20. Hardisty, D.S. et al. An evaluation of sedimentary molybdate and iron as proxies for pre fluid paleoredox conditions. *Am. J. Sc.* **318**, 527–556 (2018).
