# Supplementary material for: Transient fertilization of a post-Sturtian Snowball ocean margin with dissolved phosphate by clay minerals
Source: Nat Commun. 2023 Dec 18;14:8418. doi: 10.1038/s41467-023-44240-9 (PMC10728154; doi:10.1038/s41467-023-44240-9)
Supplement: Supplementary file 3 — Description of Additional Supplementary Files [file 41467_2023_44240_MOESM3_ESM.pdf]

Description of Additional Supplementary Files:

Supplementary Dataset 1: Carbon Systematics

Supplementary Dataset 2: Major, Trace and Rare Earth Element Analysis

Supplementary Dataset 3: Iron Speciation Analysis

Supplementary Dataset 4: Co-extraction of Iron and Phosphorus

Supplementary Dataset 5: Semi-quantitative XRD mineralogical Analysis

Supplementary Dataset 6: Iron Isotope Analysis
